# Chronic kidney disease awareness among the general population: tool validation and knowledge assessment in a developing country

**DOI:** 10.1186/s12882-022-02889-2

**Published:** 2022-07-26

**Authors:** Samar Younes, Nisreen Mourad, Jihan Safwan, Mariam Dabbous, Mohamad Rahal, Marah Al Nabulsi, Fouad Sakr

**Affiliations:** 1 School of Pharmacy, Lebanese International University, Bekaa, Lebanon; 2 School of Pharmacy, Lebanese International University, Beirut, Lebanon

**Keywords:** Chronic kidney disease, Tool validation, Public awareness, Knowledge assessment, Lebanon

## Abstract

**Introduction:**

Good knowledge and early identification of chronic kidney disease (CKD) can help in preventing disease progression in its early stages and reducing undesired outcomes. The aim of the current study was to assess the level of public knowledge about CKD, determine predictors of better knowledge, and to construct and validate a CKD knowledge scale for public health assessment and research use.

**Methods:**

A community-based cross-sectional study was conducted using an electronic self-administered questionnaire. All people living in Lebanon and being 18 years of age and above were considered eligible for recruitment. CKD knowledge was assessed by a 37-item scale that was constructed by principal component analysis and then validated. The score of the CKD knowledge scale was computed from the extracted factors. A multivariable binomial logistic regression model evaluated the sociodemographic and clinical predictors of the knowledge score.

**Results:**

A total of 1308 participants were included. The scale items converged over 9 factors with Eigenvalue greater than 1 and explaining 53.26% of the total variance, and the total scale had a high Cronbach’s alpha of 0.804. All items of the scale significantly correlated with the full scale with correlation coefficients ranging from 0.082 to 0.558. The ROC curve analysis determined an optimal cutoff point of better knowledge at 47.5 with 70.6% sensitivity and 44.2% specificity. The CKD knowledge score had a median of 51.00 (IQR 47.00–55.00). Higher knowledge score was significantly associated with old age (ORa = 1.018, 95% CI 1.006–1.030, *P* = 0.003),, occupation (ORa = 3.919, 95% CI 2.107–7.288, *P* <  0.001), and recent renal function assessment (ORa = 2.314, 95% CI 1.532–3.495, P <  0.001). However, a lower knowledge score was significantly associated with lower level of education (ORa = 0.462, 95% CI 0.327–0.653, P <  0.001).

**Conclusion:**

A reliable tool to assess public knowledge and awareness about CKD was developed and validated. The overall knowledge was good, however, important gaps in CKD awareness were detected in some areas and subpopulations. Therefore, public health stakeholders need to implement targeted CKD educational activities to minimize the disease burden.

**Supplementary Information:**

The online version contains supplementary material available at 10.1186/s12882-022-02889-2.

## Introduction

Chronic kidney disease (CKD), which affects more than 10% of the world’s population, has become a global public health crisis in recent decades. CKD is characterized by progressive decline of renal function over three months or more, which is linked to a number of risk factors. Once the kidneys have been damaged, they are unable to filter blood or perform other functions, resulting in a decrease in glomerular filtration rate (GFR) and proteinuria, which can develop to end-stage renal disease (ESRD) or kidney failure. If not treated with dialysis or a kidney transplant, ESRD is deadly and irreversible [1].

Since the significant rise in the incidence of important risk factors as diabetes mellitus (DM), hypertension (HTN), a poor diet, little or no physical exercise, and metabolic syndrome, CKD has become a global public health issue. It claimed the lives of 409,000 people in 1990 and 956,000 people in 2013. CKD as a result of DM was responsible for 46,000 deaths in 1990 and 173,000 in 2013 [2]. Kidney illnesses have recently been ranked as the world’s 12th and 17th leading causes of death and disability [3]. Around 10–13% of the general population are affected by CKD, with an estimated global population of over 500 million people [4, 5]. Low GFR was listed as the 12th greatest cause of death at the worldwide level, and the 14th largest cause of Disability-Adjusted Life-Years (DALYs) among 79 risk factors in the Global Burden of Diseases, Injuries, and Risk Factors Study (GBD) in 2013 [3, 6].

Kidney failure, cardiovascular disease (CVD), and early death are all elevated by 8 to 10 times in people with CKD [4]. Acute kidney injury, anemia, mineral and bone disorders, fractures, and hospitalizations are among the other complications [7, 8].

CKD is also associated with a significant financial burden, accounting for more than 2–3% of annual healthcare expenditures in high-income countries, despite the fact that patients with ESRD account for only 0.03% of the total population, and lower socioeconomic status is linked to a higher risk of ESRD [9]. The burden of CKD in developing countries is substantially greater due to additional hazards associated with poverty, such as infections, hazardous job, inadequate education, and poor maternal health, as well as the additional expense of screening and treatment, which must be paid directly by patients [10, 11].

The public’s awareness and understanding of CKD is a critical aspect in CKD preventive and screening programs’ success, whereby early detection and management of CKD can help prevent disease progression in its early stages [12]. Despite this, the majority of CKD cases are not detected early [13]. General knowledge of CKD, its risk factors, and individual risk and CKD status consequence and understanding are all parts of CKD awareness among patients [14].

A better rate of early identification of those with undetected/early CKD or those at risk of developing CKD may be attainable in populations with high levels of knowledge and awareness about CKD [15]. According to research conducted in both developed and developing nations, the public’s understanding of CKD and its risk factors is inadequate [16]. A recent Australian study found that participants had insufficient awareness of the kidneys’ physiological significance, with less than half correctly identifying HTN as a risk factor [16]. A study conducted in Saudi Arabia in 2010 found that only 7.1% of patients with early CKD were aware of their CKD status, and that the study cohort had poor awareness of CKD symptoms [17]. However, another recent study from Saudi Arabia using a non-validated questionnaire found that more than half of the study population correctly identified HTN and DM as CKD risk factors [15]. Another study also found a deficiency of awareness about CKD among Saudis, with respondents from higher educational and economic backgrounds having much more information and those with CKD risk factors also had a better understanding of the condition [18]. Besides, Chow et al. observed a lack of broad public knowledge on CKD and recommended future research in a population of high-risk individuals [19].

CKD is a leading cause of death and disability around the world [20]. In Lebanon, there is a paucity of data on public awareness and knowledge of CKD, as well as validated tools to assess public knowledge. This study aimed to assess the level of public knowledge about CKD and to determine predictors of better knowledge. It also aimed to construct and validate a CKD knowledge scale for public health assessment and research use.

## Methods

### Study design and participants

This was a cross-sectional study that included participants from all over Lebanon. A snowball sampling was used to collect data through an electronic self-administered questionnaire between May 2021 and December 2021. The weblink to the questionnaire was shared on Facebook, LinkedIn, and WhatsApp. Involvement of illiterate participants was encouraged to explore their knowledge and awareness about CKD, which could affect their behavior toward the disease prevention. Therefore, family members or/and caregivers were encouraged to interview their illiterate family members and record the responses on behalf of them. All people living in Lebanon and being 18 years of age and above were considered eligible for recruitment. The questionnaire included a cover letter to explain the objectives of the study. The time to complete the questionnaire was about 10 to 15 minutes. Data collection was started with a pilot study on 10 participants to explore clarity, consistency, and time needed to complete the questionnaire.

### Study instrument and outcomes

The questionnaire of the study was composed of 2 parts. The first part included general sociodemographic information relating to age, gender, area of residence, level of education, occupation, marital status, monthly income, and smoking and alcohol use. It also assessed clinical characteristics relating to present medical and family histories relating to cardiovascular, metabolic and kidney diseases. The second part was composite of specific questions about the kidneys and renal function to generate a genuine CKD knowledge scale. Knowledge of CKD involved information about various aspects of the disease that the respondents had collected over their lives through experience or education, usually pertaining to the causes of the disease and exacerbating factors, identification of symptoms and complications, and available treatment modalities. As for awareness, it goes beyond knowledge to include perceiving preventative actions, screening and check-ups, therefore, it plays an important role in disease control.

The scale was constructed by 4 pharmacotherapy specialists after a comprehensive PubMed literature review. The initial scale included 39 items and was reviewed and verified by 2 external nephrologists. The scale items included multiple choice questions about renal physiology, CKD risk factors, renal assessment, CKD screening, clinical presentation and complications, correlation of kidney disease with cardiovascular and metabolic disorder, and CKD preventive measures and treatment options (Additional file 1: Annex 1). The study outcomes were to determine the public knowledge and awareness about CKD through a valid and reliable tool, and to determine predictors of better knowledge about CKD.

### Ethical aspects

The study was approved by the Ethics and Research Committee of the School of Pharmacy at the Lebanese International University (2021RC-116-LIUSOP). The study carried no harmful risks as it was observational, and anonymity of all participants was warranted as personal identifiers weren’t traced during data collection and analysis. All participants agreed to participate and provided informed consent. Illiterate people had the informed consent read and explained to them by a caregiver or family member. Informed consent was provided electronically by ticking 3 compulsory options about understanding that participation is voluntarily and confidential, and agreeing to participate before being able to proceed to the first section of the questionnaire.

### Sample size calculation

The minimal sample size was calculated using the Centers for Disease Control and Prevention (CDC) Epi Info version 7.2.4 (population surveys). The expected frequency was kept at 50% to yield the largest minimal sample size required. Accordingly, a minimal sample of 384 participants was required to allow for adequate power of statistical analysis, and produce a 95% confidence level and an acceptable margin of error of 5%. For the scale validation, a ratio of participants to items should be at least 10:1 according to the rule of thumb [21]. Therefore, a minimal sample size of 370 was required to validate the CKD knowledge scale.

### Data analysis

Data were analyzed using IBM Statistical Package for Social Sciences (IBM SPSS) version 26.0. Descriptive statistics were used to evaluate the sociodemographic and clinical characteristics of participants. Continuous variables were reported by their mean (± standard deviation), and categorical variables were reported by their frequencies and percentages. The CKD knowledge scale responses were coded with 2 for the correct answer and 1 for wrong answers of each multiple-choice item. Factor analysis with principal component analysis was run to construct and then validate the CKD knowledge scale with varimax rotation. Factors retained in the final scale had Eigen values greater than one. Adequate Kaiser–Meyer–Olkin (KMO) measure of sampling adequacy and Bartlett’s test of sphericity were ensured. KMO and Bartlett’s indicate the suitability of the data for structure detection. The KMO measure of sampling adequacy indicates the proportion of variables’ variance that might be caused by underlying factors. Bartlett’s test of sphericity tests whether the correlation matrix is an identity matrix to confirm that the factor model is appropriate. The score of the CKD knowledge scale was computed from the extracted factors. The correlation of each scale component with the whole scale was determined by Pearson correlation. Cronbach’s alpha measured the full-scale internal consistency and reliability. The scale sensitivity and specificity were determined by receiver operating characteristic (ROC) curve analysis, with optimal cutoff point determined by J-index. The normal distribution of the final knowledge score was assessed by histogram and Shapiro-Wilk test. The score was skewed so it was dichotomized according to its median. Knowledge scores above the median were considered associated with better CKD knowledge. A multivariable binomial logistic regression model utilizing forward likelihood ratio method evaluated the sociodemographic and clinical characteristics of participants as independent variables predicting a better knowledge score as the dependent variables. Results were reported as adjusted odds ratios (ORa) with 95% confidence interval (CI). *P* values < 0.05 were considered statistically significant with an acceptable margin of error = 5%.

## Results

### Sociodemographic and clinical characteristics of participants

A total of 1308 participants were included in the study. The mean age was 37.15 (±16.24), 55.4% were females, 89.9% were Lebanese, and 33% were from Beirut. The majority of participants (61.7%) had a university degree, 49.1% were married, 33.6% were non-healthcare professionals, and most of the participants were non-smokers and non-alcoholic (52.8 and 82.6% respectively). For the monthly income, 49.2% had a family income between 500 to 1500 USD based on the official Lebanese rate of 1 USD = 1515 LBP. Greater than half of participants (61.2%) did not have a current cardiovascular or chronic kidney disease, yet their family history included HTN (57.2%), DM (45.1%), and CKD (9.5%). With respect to renal function screening assessment by a blood or urine test, 33.7% never screened their kidney function and 16.7% don’t know if they ever had the kidneys assessed. The complete sociodemographic and clinical characteristics of participants are reported in Table 1.

**Table 1:** Sociodemographic and clinical characteristics of participants

Variable	Frequency or (Mean ± S.D., range)	Percentage
**Age**	37.15 (±16.24, 18–90)	
**Age Group**		
18–30	649	49.6
31–49	311	23.8
50+	348	26.6
**Gender**		
Male	583	44.6
Female	725	55.4
**Nationality**		
Lebanese	1176	89.9
Non-Lebanese	132	10.1
**Area of residence**		
Beirut	431	33
Bekaa	189	14.4
Mount Lebanon	239	18.3
North	144	11
South	305	23.3
**Marital status**		
Single	572	43.7
Married	642	49.1
Divorced/Widowed/Separated	94	7.2
**Education status**		
Illiterate	97	7.4
Primary school	160	12.2
Secondary school	244	18.7
University	807	61.7
**Occupation**		
Student	380	29.1
Healthcare	138	10.6
Non-healthcare	439	33.6
Unemployed	274	20.9
Retired	77	5.9
**Alcoholic**		
Yes	228	17.4
No	1080	82.6
**Smoker**		
Yes	618	47.2
No	690	52.8
**Family income**		
< 500$	145	11.1
500–1500$	644	49.2
> 1500$	519	39.7
**Health insurance**		
NSSF^a^	658	50.3
COOP^b^	92	7
Private	306	23.4
None	318	24.3
**Present medical history**		
Hypertension	325	24.8
Diabetes mellitus	209	16
Hyperlipidemia	221	16.9
Chronic kidney disease	121	9.3
Cardiac disease	107	8.2
Cerebrovascular disease	26	2
None	801	61.2
**Family history**		
Hypertension	748	57.2
Diabetes mellitus	590	45.1
Hyperlipidemia	397	30.4
Chronic kidney disease	124	9.5
Cardiac disease	387	29.6
Cerebrovascular disease	55	4.2
None	276	21.1
**Last renal function assessment/ screening**		
< 6 months	225	17.2
6–12 months	129	9.9
1–2 years	133	10.2
> 2 years	162	12.4
Never been tested for renal function	441	33.7
Do not know/ remember	218	16.7
**Attended any CKD awareness presentation**		
Yes	164	12.5
No	1144	87.5

*S.D* Standard deviation, *CKD* Chronic kidney disease

^a^*NSSF* National social security fund

^b^*COOP* Cooperation healthcare fund

### Factor analysis

The factor analysis selection variable was Lebanese participants from the total sample representing the general population including Lebanese and non-Lebanese participants. After eliminating 5 variables from the initial CKD knowledge scale because they didn’t load well on any factor, the final scale included a total of 34 variables (Additional file 2: Annex 2). No variables of the final scale over correlated with each other (r > 0.9), had low communality (< 0.3), or low factor loading (< 0.3). The KMO measure of sampling adequacy was 0.881 with a significant Bartlett’s test of sphericity (*P* <  0.001). The scale items converged over 9 factors with Eigenvalue greater than 1 and explaining 53.26% of the total variance. The varimax rotated matrix of the scale components is shown in Table 2.

**Table 2:** Varimax rotated matrix of CKD knowledge scale

	Item number	Loading factor	Communalities
**Factor 1**			
Hypertension	^a^A6.1	0.676	0.504
Keep blood pressure under control	^a^A8.3	0.620	0.505
Help in maintaining blood pressure	^b^K2.5	0.612	0.564
Blood pressure monitoring	^a^A3.4	0.515	0.509
Heart diseases such as heart failure or heart attack	^a^A6.4	0.426	0.435
Affected organs: heart	^a^A5.1	0.451	0.487
**Factor 2**			
Nausea and vomiting	^b^K7.1	0.710	0.545
Loss of appetite	^b^K7.3	0.665	0.549
Keep blood sugar levels under control	^a^A8.2	0.531	0.508
Tiredness/ fatigue	^b^K7.2	0.519	0.444
Diabetes	^a^A6.2	0.469	0.550
Fever	^**b**^K7.4	0.414	0.524
**Factor 3**			
Pain killers (e.g., NSAIDs)	^b^K6.5	0.561	0.472
Fluid overload	^b^K7.5	0.503	0.482
Limit the intake of juices and soft drinks	^a^A8.1	0.472	0.475
Dialysis	^b^K9.2	0.469	0.426
Family history of CKD	^a^A6.3	0.465	0.383
Drugs	^b^K9.1	0.442	0.380
**Factor 4**			
Obesity	^a^A6.6	0.725	0.649
Excess stress	^a^A6.7	0.684	0.570
Keep body weight under control	^a^A8.4	0.549	0.537
**Factor 5**			
Produce substances that break down fats	^b^K2.6	0.765	0.632
Break down protein in the body	^b*^K2.2	0.728	0.579
Help in keeping the bones healthy	^b^K2.4	0.591	0.556
**Factor 6**			
Affected organs: lungs	^a^A5.2	0.617	0.492
Affected organs: skin	^a^A5.3	0.611	0.498
Affected organs: brain	^a^A5.4	0.603	0.614
**Factor 7**			
Produce urine	^b^K2.1	0.696	0.644
Clean blood/ filter waste products in the blood	^b^K2.3	0.621	0.522
CKD stages	^b^K4	0.465	0.455
**Factor 8**			
Renal assessment via fecal test	^b^K3.3	0.661	0.544
Number of kidneys in a healthy individual	^b^K1	0.625	0.571
**Factor 9**			
Renal assessment via urine test	^a^A3.1	0.747	0.664
Renal assessment via blood test	^a^A3.2	0.654	0.603

*NSAIDs* Nonsteroidal anti-inflammatory drugs, *CKD* Chronic kidney disease

^a^*A* Awareness

^b^*K* Knowledge

### Correlation and reliability

The scale had a high Cronbach’s alpha of 0.804. All items of the scale significantly correlated with the full scale. The correlation coefficients ranged from 0.082 to 0.558. Table 3 reports the correlation of each item of the CKD knowledge scale with the whole scale.

**Table 3:** Correlation of each item of CKD knowledge scale with the whole scale

Total CKD knowledge scale		
	Correlation	***P***-value
1. Number of kidneys in a healthy individual	0.082	0.003
2.1. Produce urine	0.242	< 0.001
2.2. Break down protein in the body	0.357	< 0.001
2.3. Clean blood/ filter waste products in the blood	0.368	< 0.001
2.4. Help in keeping the bones healthy	0.484	< 0.001
2.5. Help in maintaining blood pressure	0.570	< 0.001
2.6. Produce substances that break down fats	0.300	< 0.001
3.1. Renal assessment via urine test	0.257	< 0.001
3.2. Renal assessment via blood test	0.398	< 0.001
3.3. Renal assessment via fecal test	0.239	< 0.001
3.4. Blood pressure monitoring	0.493	< 0.001
4. CKD stages	0.181	< 0.001
5.1. Affected organs: heart	0.212	< 0.001
5.2. Affected organs: lungs	0.282	< 0.001
5.3. Affected organs: skin	0.316	< 0.001
5.4. Affected organs: brain	0.328	< 0.001
6.1. Hypertension	0.479	< 0.001
6.2. Diabetes	0.558	< 0.001
6.3. Family history of CKD	0.448	< 0.001
6.4. Heart diseases such as heart failure or heart attack	0.541	< 0.001
6.5. Pain killers (e.g., NSAIDs)	0.475	< 0.001
6.6. Obesity	0.552	< 0.001
6.7. Excess stress	0.487	< 0.001
7.1. Nausea and vomiting	0.471	< 0.001
7.2. Tiredness/ fatigue	0.565	< 0.001
7.3. Loss of appetite	0.536	< 0.001
7.4. Fever	0.362	< 0.001
7.5. Fluid overload	0.462	< 0.001
8.1. Limit the intake of juices and soft drinks	0.279	< 0.001
8.2. Keep blood sugar levels under control	0.543	< 0.001
8.3. Keep blood pressure under control	0.533	< 0.001
8.4. Keep body weight under control	0.590	< 0.001
9.1. Drugs	0.356	< 0.001
9.2. Dialysis	0.377	< 0.001

*CKD* Chronic kidney disease, *NSAIDs* Nonsteroidal anti-inflammatory drugs

### Validity measures

**Fig. 1:**
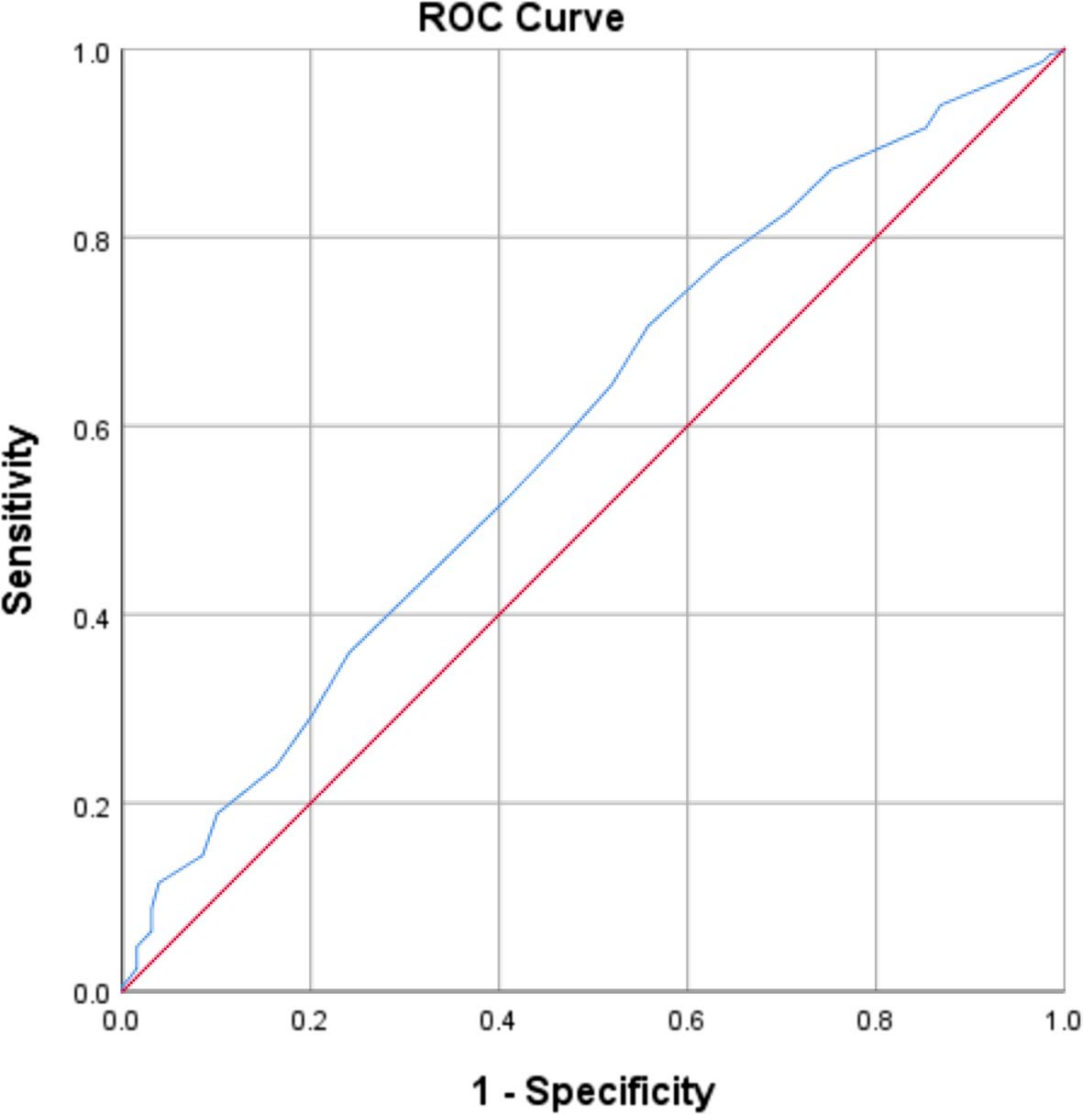
ROC curve of CKD knowledge scale

Figure 1 presents the ROC curve of the CKD knowledge scale comparing Lebanese to non-Lebanese participants from the general population. The ROC curve analysis determined an optimal cutoff point of better knowledge at 47.5 with 70.6% sensitivity and 44.2% specificity. The area under the curve was 0.597 (95% CI 0.545–0 .649, *P* <  0.001).

### Knowledge score and predictors of better knowledge

The CKD knowledge score for total correct knowledge had a median of 51.00 (IQR 47.00–55.00) with a minimum and maximum values of 38 and 70 respectively. There is a significant positive association between age and area of residence with the knowledge score. Older participants had significantly higher score (ORa = 1.018, 95% CI 1.006–1.030, *P* = 0.003). Residents of Bekaa, Mount Lebanon, and North governorates also had significantly higher scores compared to South residents (ORa = 1.623, 95% CI 1.056–2.493, *P* = 0.027); (ORa = 1.671, 95% CI 1.116–2.501, *P* = 0.013); and (ORa = 1.691, 95% CI 1.050–2.722, *P* = 0.031) respectively.

There is a significant association between the level of education and occupation with the knowledge score. A significantly lower knowledge score was associated with secondary school (ORa = 0.462, 95% CI 0.327–0.653, *P* <  0.001), primary school (ORa = 0.386, 95% CI 0.249–0.600, *P* <  0.001), and illiteracy (ORa = 0.248, 95% CI 0.145–0.422, *P* <  0.001) compared to university education. A significantly higher score was associated with being a healthcare professional (ORa = 3.919, 95% CI 2.107–7.288, P <  0.001) and a student (ORa = 2.497, 95% CI 1.585–3.934, P <  0.001) compared to unemployed participants.

Finally, there is a significant positive association between recent renal function assessment and knowledge score. Participants with a renal function assessment within the last 6 months and 1 year had significantly higher score compared to those who never tested their kidney function (ORa = 2.314, 95% CI 1.532–3.495, *P* <  0.001) and (ORa = 2.124, 95% CI 1.291–3.496, *P* = 0.003) respectively. The multivariable logistic regression of CKD knowledge score predictor is reported in Table 4.

**Table 4:** Multivariable logistic regression of CKD knowledge score predictors

Variable	ORa	***P***-value	95% CI
Age	1.018	0.003	1.006–1.030
Area of residence			
▪ Beirut vs. South	0.902	0.537	0.649–1.253
▪ Bekaa vs. South	1.623	0.027	1.056–2.493
▪ Mount Lebanon vs. South	1.671	0.013	1.116–2.501
▪ North vs. South	1.691	0.031	1.050–2.722
Level of education			
▪ Illiterate vs. University	0.248	< 0.001	0.145–0.422
▪ Primary school vs. University	0.386	< 0.001	0.249–0.600
▪ Secondary school vs. University	0.462	< 0.001	0.327–0.653
Occupation			
▪ Healthcare vs. Unemployed	3.919	< 0.001	2.107–7.288
▪ Non-healthcare vs. Unemployed	1.041	0.820	0.739–1.466
▪ Retired vs. Unemployed	1.126	0.699	0.618–2.049
▪ Student vs. Unemployed	2.497	< 0.001	1.585–3.934
Last assessment of renal function			
▪ < 6 months vs. Never	2.314	< 0.001	1.532–3.495
▪ 6–12 months vs. Never	2.124	0.003	1.291–3.496
▪ 1–2 years vs. Never	1.394	0.156	0.881–2.205
▪ > 2 years vs. Never	1.422	0.107	0.927–2.181
▪ Don’t know or remember vs. Never	0.923	0.665	0.642–1.326

*ORa* Adjusted odd’s ratio, *95% CI* 95% Confidence interval

## Discussion

The current study constructed and validated the CKD knowledge scale as a reliable tool to assess public knowledge about CKD, and promote public health awareness and research. It also assessed the level of knowledge on CKD and determined predictors of better knowledge among the Lebanese population. We found a good overall knowledge about CKD, with a significant better knowledge associated with age, area of residence, level of education, occupation, and a recent assessment of kidney function.

This research was able to develop and validate a novel CKD knowledge scale that intends to use evidence-based literature to assess the public awareness about CKD [16, 22–24]. Our findings provided evidence that the scale is valid and reliable in determining the level of knowledge on CKD especially in the Lebanese population. The nine factors in the scale had a very good internal consistency [25], and the results suggested very good reproducibility as all components correlated with the full scale with high significance. Although further research is still recommended to confirm the reproducibility and validity of the scale as the correlation coefficients of the scale’s items with the full scale were low.

A number of surveys assessed public awareness about CKD and provided validated questionnaires for this assessment. For instance, Gheewala et al. developed a validated questionnaire to assess public knowledge of CKD in Australia [16]. Furthermore, CKD knowledge questionnaires developed by Peng et al. [26] and Wei et al. [27] showed favorable construct validity, reliability, and consistency. The present study provided an additional tool of evaluation in the form of a valid scale of knowledge assessment. The construct validity was confirmed by computing the sensitivity and specificity of the scale. Our scale had a very good sensitivity supporting its use for public knowledge assessment. Nonetheless, the specificity of the scale was relatively low. This could be justified by the fact that the scale items were general and aimed at being understood and self-administered by the general population.

We found a good level of knowledge about CKD as determined by the median of the score of the knowledge scale. Our results are consistent with other findings that reported good knowledge about CKD. A study conducted in Jordan revealed that most of the participants had appropriate knowledge about CKD [28]. Moreover, nearly half of the participants in a study conducted by Tegegne et al. were well-knowledgeable on the prevention and early detection of CKD [29]. Contrary to these findings, in a study of the Australian public’s understanding of CKD, half of the participants had knowledge scores of less than the median [16]. Furthermore, Tuot et al. [30], Ng et al. [31], Agustiyowati [32], as well as, Hussain et al. [14] reported low levels of knowledge and awareness of CKD. Our results added to the literature that knowledge scores above the median or the mean could reflect substantial awareness about CKD, as our ROC curve analysis determined a lower cutoff point for better knowledge which was below the knowledge score median.

The results of our study revealed a significant positive association between age and a better CKD knowledge. Older population appear to be more knowledgeable about CKD. Maturity and more years of life experience could be behind this association. Our findings support the literature that determined better knowledge of CKD among older population. In a study conducted in Saudi Arabia, participants with a greater age showed a substantially higher CKD knowledge score [18]. The risk of cardiovascular and renal diseases increases with age and is associated with high rates of morbidity and mortality [33, 34]. Older patients may also be more concerned about their health, which could be the reason to acquire more information about chronic conditions including kidney disease to prevent or control their medical conditions.

Our findings support the literature that reported better health literacy in urban regions compared to rural areas. These findings are in line with those reported by Asmelash et al. where urban residents were 2.21 times more knowledgeable about CKD when compared to rural residents [35]. Likewise, higher mean knowledge score was observed among urban participants when compared to rural participants in a study conducted in Tanzania [36]. The above results showed better knowledge in Bekaa, Mount Lebanon, and North governorates compared to the South of Lebanon. The reason for this is not fully understood. Though it could be explained by that the South population are less concerned about their health and medical conditions. This is reflected by recent national reports that showed lower immunization rates and acceptance of COVID-19 vaccination in this region [37].

The results of the current study are consistent with the normal trend of having a better health literacy with higher level of education [38]. Our results determined that having a university degree is an important predictor of knowledge about CKD. For instance, participants with a higher education level had a higher CKD knowledge score, according to Chow et al. [23]. Also, in a study conducted by Stanifer et al., results demonstrated that participants who had completed secondary or post-secondary school had higher mean scores than those who had just completed primary school or had no education [36]. In fact, highly educated populations are reportedly more knowledgeable about various medical and non-medical conditions [39], and this research confirms that CKD is not an exception. Healthcare providers were also found to have significantly better knowledge; this was in line with findings in Ethiopia which showed that care provider professionals had enough knowledge and practice towards CKD [40]. This is not surprising, as healthcare professionals are expected to be competent with the basics of all medical conditions including nephrology. Moreover, students appear to have better knowledge about CKD. This could be justified by higher digital literacy among this subpopulation and the wider access they have to medical information [41, 42].

People who are more concerned about their health conditions appear to have a better awareness about CKD. We found a significantly better knowledge in this context among those who screened or assessed their renal function lately within a maximum of 1 year. This subpopulation is assumed to have better attitude toward selfcare and awareness, which is associated with routine medical checkups and better knowledge relating to various chronic conditions including CKD [43].

### Implications for practice

This study was able to generate and validate a reliable assessment tool for public knowledge and awareness about CKD. Although a good level of knowledge was determined, important gaps around CKD awareness were found among younger, less educated, and unemployed subpopulations. Similar gaps appear to exist in rural areas and among people who are less concerned about their medical wellbeing. The findings of this research provide insight for public health stakeholders to stipulate more attention, education, and healthcare for those populations in order to minimize the disease burden.

### Strengths and limitations

This study has several strengths. It provides public health researchers and healthcare providers with a reliable, consistent, and valid tool to assess public awareness about CKD. This tool could be modified and adapted in future research to assess public awareness about additional medical conditions. The sample size was considerably large and thus allowed for a high power of statistical analysis. On the other hand, the limitations of the study include a possible selection bias as a result of the snowball sampling. However, the risk of this bias is minimized as the sample included participants from districts all over Lebanon. Furthermore, a possible information bias that could be related to self-administration of the questionnaire cannot be precluded. Though it is believed that this kind of bias is also minimized since the questionnaire was in simple lay language and didn’t include any medical jargons. Another limitation is related to the cross-sectional design of the study, which cannot determine temporality. Moreover, the sensitivity and specificity of the scale were relatively low, and the study sample included relatively young participants. Therefore, additional research is recommended to include older participants to confirm the validity of the scale. Finally, although this novel tool of assessment appears to be valid and reliable, the validation involved exploratory factor analysis only. Further research is suggested in this context to confirm the factor analysis and validity measures of this assessment and research tool.

## Conclusion

In conclusion, the current study developed and validated a reliable tool to assess the general public’s knowledge and awareness about CKD. Findings revealed that Lebanese knowledge was good in general and was associated with age, area of residence, level of education, occupation, and a recent assessment of kidney function. Nonetheless, significant gaps in CKD awareness were observed in some areas and subpopulations. Therefore, responsible organizations should make an extra effort to raise community awareness and implement targeted CKD educational activities to improve the early detection and management of CKD.

## Supplementary Information

**Additional file 1: Annex 1.** The study questionnaire including the initial CKD knowledge scale.

**Additional file 2: Annex 2.** The final validated CKD knowledge scale.

## Data Availability

The datasets used and/or analyzed during the current study are available from the corresponding author on reasonable request.

## Abbreviations

CKD: Chronic kidney disease
GFR: Glomerular filtration rate
ESRD: End-stage renal disease
DM: Diabetes mellitus
HTN: Hypertension
DALYs: Disability-Adjusted Life-Years
GBD: Global Burden of Diseases, Injuries, and Risk Factors Study
CVD: Cardiovascular disease
CDC: Centers for Disease Control and Prevention
SPSS: Statistical Package for Social Sciences
KMO: Kaiser–Meyer–Olkin
ROC: Receiver operating characteristic
ORa: Adjusted odds ratios
CI: Confidence interval
NSAIDs: Nonsteroidal anti-inflammatory drugs
