# Supplementary material for: Chronic kidney disease awareness among the general population: tool validation and knowledge assessment in a developing country
Source: BMC Nephrol. 2022 Jul 26;23:266. doi: 10.1186/s12882-022-02889-2 (PMC9316863; doi:10.1186/s12882-022-02889-2)
Supplement: Supplementary file 1 — Additional file 1: Annex 1. The study questionnaire including the initial CKD knowledge scale. [file 12882_2022_2889_MOESM1_ESM.docx]

**Annex 1: The study questionnaire including the initial CKD knowledge scale**

**PART A: DEMOGRAPHIC DATA**

| A1 | **Gender** | 1. Male 2. Female |
| --- | --- | --- |
| A2 | **Age** |  |
| A3 | **Nationality** | 1. Lebanese 2. Non-Lebanese |
| A4 | **Marital Status** | - - - 1. Single       2. Married       3. Divorced/Widowed |
| A5 | **Educational Status** | 1. No education 2. Primary School 3. Secondary School 4. University and above |
| A6 | **Occupation** | 1. Student 2. Healthcare 3. Non-Healthcare 4. Unemployed 5. Retired 6. Other/Unknown |
| A7 | **Alcoholic** | 1. Yes 2. No |
| A8 | **Smoker** | 1. Yes 2. No |
| A9 | **Family income** | 1. <500$ 2. 500-1500$ 3. > 1500$ |
| A10 | **Health insurance** | 1. NSSF 2. COOP 3. Private 4. None |
| A11 | **Do you have any of these medical conditions? (Please tick all that apply)** | 1. Hypertension 2. Diabetes mellitus 3. Hyperlipidemia 4. Chronic kidney disease 5. Cardiac disease 6. Cerebrovascular disease 7. None of the above |
| A12 | **Do you have family history (first degree relative) of these medical conditions? (Please tick all that apply)** | 1. Hypertension 2. Diabetes mellitus 3. Hyperlipidemia 4. Chronic kidney disease 5. Cardiac disease 6. Cerebrovascular disease 7. None of the above |
| A13 | **When was the last time that you have been tested for your renal function?** | 1. < 6 months ago 2. 6-12 months ago 3. 1-2 years ago 4. > 2 years ago 5. Never been tested for renal function 6. Do not know/remember |
| A14 | **Have you ever attended a seminar or workshop about chronic kidney disease?** | 1. Yes 2. No |

**PART B: PLEASE CHOOSE THE CORRECT ANSWER FOR THE FOLLOWING QUESTIONS REGARDING YOUR KNOWLEDGE AND PERCEPTION OF KIDNEY DISEASE:**

| **B1. What is the number of kidneys in a normal individual?**   1. 1 2. 2 3. 3 4. 4 | | | | | | |
| --- | --- | --- | --- | --- | --- | --- |
| **B2. What is/are the function(s) of the kidney?** | | | | | | |
| 1. Makes urine | Yes | No | | | | Don’t know |
| 1. Breaks down protein in the body | Yes | | No | | | Don’t know |
| 1. Cleans blood/filters waste products in the blood | Yes | | No | | | Don’t know |
| 1. Helps in keeping the bones healthy | Yes | | No | | | Don’t  know |
| 1. Helps in maintaining blood pressure | Yes | | No | | | Don’t know |
| 1. Produces substances that break down fats | Yes | | No | | | Don’t know |
| **B3. The health of the kidneys can be determined by:** | | | | | | |
| 1. A urine test | Yes | | | No | | Don’t know |
| 1. A blood test | Yes | | | No | | Don’t know |
| 1. A fecal test | Yes | | | No | | Don’t know |
| 1. Blood pressure monitoring | Yes | | | No | | Don’t know |
| **B4. What is chronic kidney disease?**   1. A disease where the kidneys slowly stop working 2. A disease where a person urinates too much 3. A disease where there are a lot of stones in the kidney 4. A disease where there is cancer in the kidney | | | | | | |
| **B5. How many stages are there in chronic kidney disease?**   1. Two stages 2. Three stages 3. Four stages 4. Five stages | | | | | | |
| **B6. Which age group is at greatest risk for developing chronic kidney disease?**   1. Newborn babies 2. Teenagers 3. Young adults between the ages of 25-35 years 4. Older adults over 50 years old | | | | | | |
| **B7. What organs can be affected in patients with chronic kidney disease?** | | | | | | |
| 1. Heart | Yes | | | | No | Don’t know |
| 1. Lungs | Yes | | | | No | Don’t know |
| 1. Skin | Yes | | | | No | Don’t know |
| 1. Brain | Yes | | | | No | Don’t know |
| **B8. What is the leading cause of death in a person who has chronic kidney disease?**   1. Heart disease 2. Bone disease 3. Liver disease 4. Kidney disease | | | | | | |
| **B9. What are the risk factors for chronic kidney disease?** | | | | | | |
| 1. Hypertension | Yes | | | | No | Don’t  know |
| 1. Diabetes | Yes | | | | No | Don’t  know |
| 1. Family history of CKD | Yes | | | | No | Don’t  know |
| 1. Being female | Yes | | | | No | Don’t  know |
| 1. Heart diseases such as heart failure or heart attack | Yes | | | | No | Don’t  know |
| 1. Pain killers (e.g. NSAIDS) | Yes | | | | No | Don’t  know |
| 1. Obesity | Yes | | | | No | Don’t  know |
| 1. Excess stress | Yes | | | | No | Don’t  know |
| **B10. What are the signs and symptoms that people with advanced chronic kidney disease might have?** | | | | | | |
| 1. Nausea/vomiting | Yes | | | | No | Don’t  know |
| 1. Tiredness/fatigue | Yes | | | | No | Don’t  know |
| 1. Loss of appetite | Yes | | | | No | Don’t  Know |
| 1. Fever | Yes | | | | No | Don’t  know |
| 1. Fluid overload (excess water in the body) | Yes | | | | No | Don’t  know |
| **B11. Chronic kidney disease can be prevented by which of the following measures?** | | | | | | |
| 1. Limit the intake of juices and soft drinks | Yes | | | | No | Don’t know |
| 1. Keep blood sugar levels under control | Yes | | | | No | Don’t know |
| 1. Keep blood pressure under control | Yes | | | | No | Don’t know |
| 1. Keep weight under control | Yes | | | | No | Don’t know |
| **B12. How can chronic kidney disease be treated?** | | | | | | |
| 1. Drugs | Yes | | | | No | Don’t  know |
| 1. Dialysis | Yes | | | | No | Don’t  know |
| 1. Transplantation | Yes | | | | No | Don’t  know |
