# Supplementary material for: Chronic kidney disease awareness among the general population: tool validation and knowledge assessment in a developing country
Source: BMC Nephrol. 2022 Jul 26;23:266. doi: 10.1186/s12882-022-02889-2 (PMC9316863; doi:10.1186/s12882-022-02889-2)
Supplement: Supplementary file 2 — Additional file 2: Annex 2. The final validated CKD knowledge scale. [file 12882_2022_2889_MOESM2_ESM.docx]

**Annex 2: The final validated CKD knowledge scale**

| **B1. What is the number of kidneys in a normal individual?**   1. 1 2. 2 3. 3 4. 4 | | | | | | |
| --- | --- | --- | --- | --- | --- | --- |
| **B2. What is/are the function(s) of the kidney?** | | | | | | |
| 1. Makes urine | Yes | No | | | | Don’t know |
| 1. Breaks down protein in the body | Yes | | No | | | Don’t know |
| 1. Cleans blood/filters waste products in the blood | Yes | | No | | | Don’t know |
| 1. Helps in keeping the bones healthy | Yes | | No | | | Don’t  know |
| 1. Helps in maintaining blood pressure | Yes | | No | | | Don’t know |
| 1. Produces substances that break down fats | Yes | | No | | | Don’t know |
| **B3. The health of the kidneys can be determined by:** | | | | | | |
| 1. A urine test | Yes | | | No | | Don’t know |
| 1. A blood test | Yes | | | No | | Don’t know |
| 1. A fecal test | Yes | | | No | | Don’t know |
| 1. Blood pressure monitoring | Yes | | | No | | Don’t know |
| **B4. How many stages are there in chronic kidney disease?**   1. Two stages 2. Three stages 3. Four stages 4. Five stages | | | | | | |
| **B5. What organs can be affected in patients with chronic kidney disease?** | | | | | | |
| 1. Heart | Yes | | | | No | Don’t know |
| 1. Lungs | Yes | | | | No | Don’t know |
| 1. Skin | Yes | | | | No | Don’t know |
| 1. Brain | Yes | | | | No | Don’t know |
| **B6. What are the risk factors for chronic kidney disease?** | | | | | | |
| 1. Hypertension | Yes | | | | No | Don’t  know |
| 1. Diabetes | Yes | | | | No | Don’t  know |
| 1. Family history of CKD | Yes | | | | No | Don’t  know |
| 1. Heart diseases such as heart failure or heart attack | Yes | | | | No | Don’t  know |
| 1. Pain killers (e.g. NSAIDS) | Yes | | | | No | Don’t  know |
| 1. Obesity | Yes | | | | No | Don’t  know |
| 1. Excess stress | Yes | | | | No | Don’t  know |
| **B7. What are the signs and symptoms that people with advanced chronic kidney disease might have?** | | | | | | |
| 1. Nausea/vomiting | Yes | | | | No | Don’t  know |
| 1. Tiredness/fatigue | Yes | | | | No | Don’t  know |
| 1. Loss of appetite | Yes | | | | No | Don’t  know |
| 1. Fever | Yes | | | | No | Don’t  know |
| 1. Fluid overload (excess water in the body) | Yes | | | | No | Don’t  know |
| **B8. Chronic kidney disease can be prevented by which of the following measures?** | | | | | | |
| 1. Limit the intake of juices and soft drinks | Yes | | | | No | Don’t know |
| 1. Keep blood sugar levels under control | Yes | | | | No | Don’t know |
| 1. Keep blood pressure under control | Yes | | | | No | Don’t know |
| 1. Keep weight under control | Yes | | | | No | Don’t know |
| **B9. How can chronic kidney disease be treated?** | | | | | | |
| 1. Drugs | Yes | | | | No | Don’t  know |
| 1. Dialysis | Yes | | | | No | Don’t  know |
